# Professor Hohl’s Case of Double Uterus, with Twins, and Placenta Praevia

**Published:** 1854-11

**Authors:** 


*Professor Hohl's Case of Double Uterus, with Twins, and Placenta Praevia.*—A delicate woman, aged 30, was subject, during the first three months of her second pregnancy, to periodically recurring attacks of uterine hemorrhage, which, although easily checked at that time, returned more violently in the seventh month. At this period her abdomen was found very much distended at both sides, but level in the central region extending from the umbilicus to the symphysis pubis. Percussion yielded a tympanitic sound in this hollow, and a dull sound on either side. An inch and a half above the pubis the uterus could be felt distinctly, through the parietes, dividing into two parts, of which the one on the right side was largest. Both were convex on their internal, and somewhat concave on their external surfaces; and each resembled in appearance the normal gravid uterus at the full time. In each, the fœtal heart could be distinctly heard on auscultation, and the form of a child easily felt by external tactile examination. On internal exploration, the vagina was found quite normal; and at its roof the cervix was felt, short and broad, and having two ora uteri, through both of which the presenting parts of the children could be distinguished. Over the right os uteri was situated one of the placentæ, and a portion of the other projected from the left os. On account of the violent uterine hemorrhage, Hohl induced premature labor, and delivered the children by turning. The right placenta was spontaneously detached, but that on the left side adhered so firmly, that it had to be artificially separated. The twins, who weighed three pounds, died almost immediately after their birth. Subsequent examinations of the uterus, with the sound and the finger, confirmed the correctness of the original diagnosis.—*Edinburgh Journ. Med. Science,* from *Deutsche Klinik. I.* 1853.

*Case of total Inversion of the Uterus, in which extirpation of the entire organ was successfully practised.* By E. Geddings, M. D., Professor of Surgery, &c., Medical College of the State of South Carolina.—On the 16th of May, 1854, I was requested, by Dr. A. P. Pelzer, to meet him in consultation, in the case of a negro woman belonging to Mr. Robert White, in King street. On my arrival, Dr. Pelzer called my attention to a large pyriform tumor, equal in magnitude to a fœtal head at the full term, which, proceeding from within the vagina, hung pendant between the thighs. This tumor was large and rounded below, but contracted into a rather thick pedicle above, which could be traced about three-fourths of an inch within the vulva, at which point its contour was surrounded by a kind of *cul de sac*, beyond which the finger could not be passed. Its whole surface was covered by a rough, thickened mucous membrane, abraded and ulcerated on many points, considerably inflamed, and disposed to bleed when roughly handled. In the general aspect, it bore a strong resemblance to a case of prolapsus of the uterus of long standing; but the uniform roundness of the most dependent part, together with the absence of the os tincæ, served at once to convince us that it was of a totally different nature.

The first supposition that presented itself to my mind was, that it might be a case of prolapsus of the bladder, of such long duration that the walls of the organ had become very much thickened, and otherwise altered in texture. But on introducing the catheter, and passing my index finger around the neck of the tumor within the vulva, I was enabled readily to discover that it was a case of complete inversion, with extensive hypertrophy of the uterus, of ancient date. The orifice of the urethra was but little removed from its normal position: and in passing my finger up, on the posterior and lateral aspects of the neck of the tumor, as far as the reflected walls of the vagina would allow it to reach, I could distinctly discover the elastic feel imparted by the convolutions of the small intestines, which rested on the partially inverted walls of the vagina.

How long the inversion had existed could not be satisfactorily ascertained; but as there is reason to suspect that the accident must have occurred at the period of her last delivery, an approximative conclusion may be drawn from the fact that her youngest child, a daughter, was present, and had the appearance of a person of from eighteen to twenty years of age. The report of the woman herself was, that she had been greatly annoyed by the tumor for many years, but had generally been enabled, by partially forcing it up into the vagina, and sustaining it there by means of a T bandage, to pursue her ordinary avocations. Latterly it had increased so much in size as to render this impracticable; and at the period of our visit, any attempt at replacement, however partial, was productive of excruciating pain. She was, besides, suffering so much from engorgement and inflammation of the inverted organ, that, considering this, together with the partial and uncertain benefit likely to accrue from any merely palliative treatment, it became a serious question how we could most readily and efficiently relieve our patient.

Reflecting on all the circumstances of the case, it occurred to me that excision of the entire inverted organ presented a rational prospect of relieving, not only the present suffering, but also the cause of much future annoyance. The vagina being also partially inverted, the danger of such an operation was materially diminished; inasmuch as we would, in consequence of that condition, be enabled to excise the entire mass by cutting through the vaginal walls, thus leaving the substance of the uterus untouched.

Dr. Pelzer concurring with me, I seized the neck of the tumor as high up as possible, between the thumb and index finger, and manipulating in such a manner as to satisfy myself that it contained none of the convolutions of the intestines, I proceeded to include it in a strong ligature, for the two-fold purpose of preventing the protrusion of the intestines and obviating any serious hæmorrhage. The neck of the tumor was then cut through, a little below the ligature, with a single swipe of a probe-pointed bistoury.

The operation was exceedingly simple and easy; was attended with no great pain; and, as may be supposed, was executed in a few seconds.

The after-treatment presented no features of particular interest; and the case progressed so favorably that, after a few days, I was enabled to discontinue my visits, leaving the patient in the hands of Dr. Pelzer; who, in a short time, transferred her to Prof. Frost, the family physician, who, at the period of our attendance, was absent from the city. She speedily recovered, and, as I understand, has since done well.

On making a section of the tumor, it was found to present a solid, homogeneous mass, of a greyish white texture and fibrous appearance. The whole cavity formed by the inversion of the walls had become obliterated by adhesions between the opposing peritoneal surfaces; but the point of junction between the vagina and the contour of the cervix could be distinctly recognized; the incision, as stated above, having passed through the walls of the vagina.

Partial and complete extirpation of the uterus, for various objects,—inversion, prolapsus, carcinomatus, and other degenerations of its structure,—has been so often practised, that the simple operation, the description of which I have detailed, possesses no claims to interest in point of novelty; yet it has some value as affording an additional instance to prove that, under similar circumstances, the unfortunate victims of a displacement so deplorable, may often be relieved of much suffering and inconvenience. It might be interesting to collect full references to the numerous cases in which extirpation has been practised on account of inversion; but, as I have not time to execute the task, I must content myself with this brief and imperfect exposition of a single case.—*Charleston Med. Jour.*

